# Stochastic mathematical model for the spread and control of Corona virus

**DOI:** 10.1186/s13662-020-03029-6

**Published:** 2020-10-14

**Authors:** Sultan Hussain, Anwar Zeb, Akhter Rasheed, Tareq Saeed

**Affiliations:** 1 Department of Mathematics, COMSATS University Islamabad, Abbottabad Campus, Abbottabad, 22060 Khyber Pakhtunkhwa Pakistan; 2 Department of Mathematics, King Abdulaziz University, Jeddah, 41206 Saudi Arabia

**Keywords:** COVID-19 epidemic, Stochastic process, Stability, Unique strong solution, Poisson process

## Abstract

This work is devoted to a stochastic model on the spread and control of corona virus (COVID-19), in which the total population of a corona infected area is divided into susceptible, infected, and recovered classes. In reality, the number of individuals who get disease, the number of deaths due to corona virus, and the number of recovered are stochastic, because nobody can tell the exact value of these numbers in the future. The models containing these terms must be stochastic. Such numbers are estimated and counted by a random process called a Poisson process (or birth process). We construct an SIR-type model in which the above numbers are stochastic and counted by a Poisson process. To understand the spread and control of corona virus in a better way, we first study the stability of the corresponding deterministic model, investigate the unique nonnegative strong solution and an inequality managing of which leads to control of the virus. After this, we pass to the stochastic model and show the existence of a unique strong solution. Next, we use the supermartingale approach to investigate a bound managing of which also leads to decrease of the number of infected individuals. Finally, we use the data of the COVOD-19 in USA to calculate the intensity of Poisson processes and verify our results.

Disease COVID-19, named after the attack of coronavirus in China at the end of 2019, spread world wide and killed more than 0.6 million individuals in initial eight months. This virus transmits person to person through respiratory droplets produced when an infected person coughs or sneezes. Infected droplets land in the noses and mouths of people who are nearby or possibly are inhaled into the lungs. It also spreads through touching a surface or object that has the virus on it and then touching your own mouth, nose, or possibly your eyes. It infects the respiratory system, and the infected person faces fever, cough, shortness of breath, and breathing difficulties. The infection and the onset of symptoms ranges from one to fourteen days. An infectious person shows symptoms within five to six days. To prevent the infection spread, one needs regular hand washing, covering mouth and nose when coughing and sneezing, and avoiding contact with effected individuals.

Mathematical modeling is a tool to study the structure of spread and control of various infectious diseases. Mathematical models have potential to educate persons about the control techniques of such diseases. It can also be used to predict the expected number of patients in the future under any controlling strategy and to set their goals. Fundamental work is done by researchers to model viral diseases and used by policy makers to control them. For example, in 2009, Pang et al. [1] considered dynamical behavior of a Hepatitis B virus transmission model with vaccination, and Zou et al. [2] discussed modeling the transmission dynamics and control of Hepatitis B virus in China. These models were implemented in China to study the number of HBV patients and to control HBV virus. To understand the spread and control of coronavirus, Chen et al. [3] proposed a mathematical model to understand the transmissibility of coronavirus. Zhou et al. [4] studied pneumonia outbreak associated with coronavirus, whereas Li et al. [5] discussed the early transmission dynamics in Wuhan, China. Huang et al. [6] provided clinical features of the patients infected with coronavirus, whereas Chan et al. [7] discussed familywise transmission of the novel coronavirus. Wu et al. [8] provided newscasting and forecasting of the COVID-19 outbreak in Wuhan, China, through modeling. Zhao et al. [9] have used the model approach to estimate the unreported number of COVID-19 in China in the first half of January 2020. Chen et al. [3] proposed a mathematical model for simulating the transmission of this virus in Wuhan. Ivorra et al. [10] developed and implemented a mathematical model on the spread of COVID-19 using data from China. Sameni [11] studied the epidemic patterns of coronavirus through a mathematical model. Kochańczyk et al. [12] studied constant and time-dependent contact rates of COVID-19 pandemic. For more detail on mathematical modeling of such viral disease, we refer the readers to [13–22]. Besides the integer-order models, fractional calculus and stochastic differential equations play an important role in the epidemic models; see [23–26]. A Poisson process is a random process that counts the number of occurrences of certain events that happen at certain rate called the intensity of the Poisson process. For more detail on this process, we refer the readers to [27].

In this work, we study a stochastic model on the spread and control of coronavirus in which the total population of an infected area is divided into susceptible, infected, and recovered classes. Generally, the exact number of individuals who get the disease, the number of deaths due to coronavirus, and the number of recovered are random and unknown, and thus these numbers are stochastic. Any model containing these terms must be stochastic. These numbers are estimated and counted by a Poisson process. We construct an SIR type model in which the above numbers are stochastic and counted by a Poisson process. To understand the flow and prevent of coronavirus in a better way, we first study the stability of the corresponding deterministic model, calculate the unique nonnegative strong solution, and investigate an inequality managing of which leads to control of the virus. After this, we pass to the stochastic model and show the existence of a unique strong solution. Next, we use the supermartingale approach to investigate a bound managing of which also leads to decrease of the number of infected individuals. Finally, we use COVID-19 data from USA (April 1 to July 19, 2020) to calculate the intensity of Poisson processes and verify our results.

The rest of the paper is organized as follows. In Sect. 2, we give a deterministic model, discus the stability, calculate solution of the model, and investigate an inequality managing of which leads to control the virus. In Sect. 3, we pass to a stochastic model and show the existence and uniqueness of a nonnegative strong solution. Next, we use COVID-19 data from USA, calculate the parameters of the Poison process, verify our results, and sketch the number of effected individuals. To control the virus, this work provides useful information to policy makers. Moreover, the results can be used to predict the number of patients in any future time to set the future goals.

## Deterministic model and stability

In this section, we consider an SIR deterministic model on the spread and control of coronavirus, discuss the local and global stability of the model and the reproductive number, calculate the unique strong solution, which can be used to calculate the future number of infected individuals, and investigate an inequality depending on some parameters of the model and the number of susceptible individuals, which would be managed to control the virus and to set the future goals.

**Figure 1:**
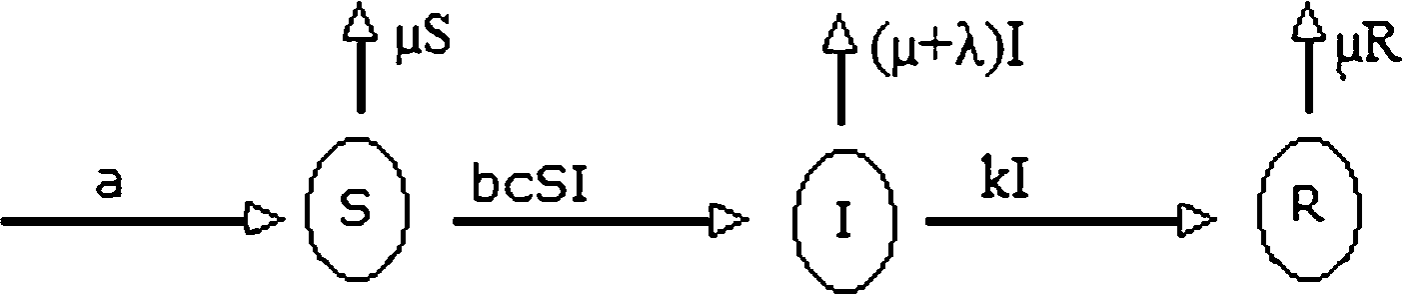
Figure shows the flow and control of the coronavirus

Let us divide the total population in three categories, susceptible, infected, and recovered individuals, and denote by $S=S(t), t\geq 0$, the number of susceptible individuals, $I=I(t)$ the number of infected individuals, and by $R=R(t)$ the number of recovered individuals. The flow and control are shown in the Fig. 1. The mathematical model on these classes is expressed by the following system of differential equations:

1 $$ \begin{aligned}& d S = (a-\mu S-bc SI )\,dt,\quad S(0)\geq 0, \\ &dI =I (bcS-\mu -k-\lambda )\,dt,\quad I(0)\geq 0, \\ &dR =(kI-\mu R)\,dt,\quad R(0)\geq 0, \end{aligned} $$

where *a* is the rate of new born and migrated individuals, *b* is the transmission rate from susceptible to infected, *c* is the contact rate of susceptible with infected, *μ* is the natural death rate, *k* is the recovery rate, and *λ* is the death rate of infected class due to virus.

In the following result, we discuss the local and global stability of the model and calculate the reproductive number $R_{0}$.

### Theorem 1

*The proposed model is locally asymptotically stable at the free virus equilibrium point* $P_{1}$ *if the reproductive number* $R_{0}<1$, *whereas it is globally asymptotically stable at the positive virus point* $P_{2}$ *if* $R_{0}>1$, *where* $R_{0}=\frac{abc}{\mu (\mu +k+\lambda )}$.

### Proof

The free virus equilibrium point is $P_{1} (\frac{a}{\mu },0,0 )$, whereas the positive virus point is $P_{2} (\frac{\mu +k+\lambda }{bc},\frac{a}{\mu +k+\lambda }- \frac{\mu }{bc},\frac{ka}{\mu (\mu +k+\lambda )}-\frac{k}{bc} )$. The Jacobian matrix of system (1) is

$$ J= \begin{pmatrix} -\mu -bcI & -bcS & 0 \\ bcI & bcS-\mu -k-\lambda & 0 \\ 0 & k & -\mu \end{pmatrix}. $$

Putting the point $P_{1}$ in the Jacobean matrix *J* gives the eigenvalues $\lambda _{1}=\lambda _{2}=-\mu $ and $\lambda _{3}=\frac{abc}{\mu }-(\mu +k+\lambda )$. Putting $\lambda _{3}<0$, we get the reproductive number $R_{0}=\frac{abc}{\mu (\mu +k+\lambda )}$. Thus all the eigenvalues are negative if and only if $R_{0}<1$, and hence the model is locally asymptotically stable.

Similarly, at point $P_{2}$, all the eigenvalues are negative if $R_{0}>1$. Hence the proposed model is globally stable if $R_{0}>1$. □

Next, we come to the nonnegative unique strong solution of the model, formula for calculating the future number of infected individuals, and an inequality that would be managed to control the virus.

### Theorem 2

*Solution of model* (1) *is nonnegative*, *and the number* $I(t)$ *decreases if the transmission function* $bcS$ *satisfies the upper bound*

$$ bcS< \mu +k+\lambda. $$

### Proof

As coefficients in the model are locally Lipschitz, the solution of the model is strong and unique (see Arnold [28]). Using integrating factors, we can express the solution of the proposed model as

2 $$\begin{aligned} & S(t)=e^{-\int _{0}^{t}(\mu +bc I(v))\,dv} \biggl[S(0)+a \int _{0}^{t}I(v)e^{ \int _{0}^{v}(\mu +bc I(u))\,du}\,dv \biggr], \\ &I(t)=I(0)e^{\int _{0}^{t}(bcS(v)-\mu -k-\lambda )\,dv}, \\ &R(t)=e^{-\mu t} \biggl[R(0)+k \int _{0}^{t}I(v)e^{\mu v}\,dv \biggr] . \end{aligned}$$

From solution (2) we first observe that $I(t)$ is nonnegative, and using this, we find that $S(t)$ and $R(t)$, are also nonnegative for all *t*.

The future value of the infected number can be calculated from the expression of $I(t)$.

For the second part, we put the exponent $bcS-\mu -k-\lambda $ into $I(t)$ less than zero and get the answer. □

Note that the product *bc* can be easily calculated by using past information in the expression of $I(t)$.

## Stochastic model formulation

In this section, we pass from deterministic to stochastic model as the number of infected population, deaths due to virus, and recovered individuals are generally not deterministic. These numbers are random and can be counted by a Poisson process. To do this, let us consider Ω a sample space, *F* a sigma algebra, $F_{t}$ a filtration of *F*, and *P* a probability measure. On a filtered probability space $(\Omega, F,F_{t}, P)_{t\geq 0}$, we consider three classes, the susceptible class $S=(S(t))_{t\geq 0}$, the infected individuals $I=(I(t))_{t\geq 0}$, the recovered class $R=(R(t))_{t\geq 0}$, and three independent Poisson processes $N_{t}$, $M_{t}$, and $K_{t}$ with intensities *λ*, *γ*, and *η*, respectively. Let $M_{t}$ be the number of individuals who got disease in the time interval $(0,t]$ due to *I*, $N_{t}$ be the number of deaths, and $K_{t}$ be the number of recovered in this interval. We replace the transmission term $\int _{0}^{t}bcS(v) \,dv$ in a deterministic form by a Poisson random variable $M_{t}$, the death term $\int _{0}^{t}(\mu +\lambda )I(v)\,dv$ by $N_{t}$, and the recovery term $\int _{0}^{t}kI(v)\,dv$ by $K_{t}$. For simplicity, we drop the natural death rate *μ* from $I(t)$ as it can be added in the number $N_{t}$.

**Figure 2:**
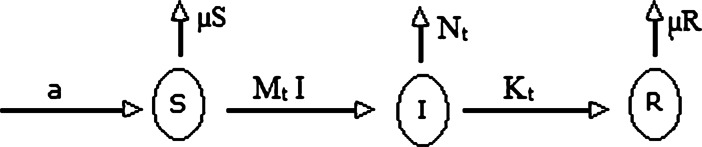
Figure shows the flow and control of coronavirus

The flow in the model is shown in Fig. 2.

The sizes of *S*, *I*, and *R* can be formulated by the system of stochastic equations (see Asmussen and Albrecher [29]) as

3 $$\begin{aligned} &S =S(0)+at-(\mu S)t-M_{t}I,\quad S(0)\geq 0, \\ &I =I(0)+ M_{t}I -N_{t}-K_{t},\quad I(0)\geq 0, \\ &R =R(0)+K_{t}-(\mu R)t,\quad R(0)\geq 0, \end{aligned}$$

with bounds

$$ 0\leq M_{t}\leq S \quad\text{and}\quad 0\leq N_{t}+K_{t}\leq I. $$

Generally, a Poison process is right continuous with left-hand limits (see Lamberton and Lapeyre [27]), and therefore the increment △ in a Poisson random process can be given as

$$ \triangle M_{t}= M_{t}-M_{t-}= \textstyle\begin{cases} 1 & \text{if there is a jump,} \\ 0 & \text{if there is no jump}, \end{cases} $$

and the derivative of the Poisson process coincides with the increment (see Lamberton and Lapeyre [27]). Thus the Poisson random processes $M_{t}$, $N_{t}$, and $K_{t}$ must satisfy

$$ \triangle M_{t}=dM_{t},\qquad \triangle N_{t}=dN_{t}\quad \text{and}\quad \triangle K_{t}=dK_{t}. $$

Since *μ* is the natural death rate, using the later expressions, the differential form of system (3) is (see Shreve [30] in case of assets modeling)

4 $$\begin{aligned} &d S =(a-\mu S)\,dt - \mathrm{Id}M_{t}, \\ &dI =\mathrm{Id}M_{t}-d(N_{t}+K_{t}), \\ &dR =d K_{t}-\mu R\,dt. \end{aligned}$$

Since most of the data is available daywise, so if *t* is time in days, then if $X_{i}$, $Y_{i}$, and $Z_{i}$, $i=1,2,\ldots $ , denote the numbers of infected individuals, deaths, and recovered individuals, respectively, at the *i*th day since the starting day $t_{0}=0$, then the Poisson processes $M_{t}$, $N_{t}$, and $K_{t}$ satisfy

5 $$ M_{t}=\sum_{i=1}^{t}X_{i},\qquad N_{t}=\sum_{i=1}^{t}Y_{i} \quad\text{and}\quad K_{t}=\sum_{i=1}^{t}Z_{i}, $$

where the random variables $X_{i}$, $Y_{i}$, and $Z_{i}$ are independent and identically distributed with uniform distribution.

If *t* is time in days, then $M_{t}$, $N_{t}$, and $K_{t}$ satisfy

$$ d M_{t}= M_{t}-M_{t-}= \textstyle\begin{cases} X_{i} & \text{if there is a jump at time $t$,} \\ 0 & \text{if there is no jump at time $t$}, \end{cases} $$

where

6 $$ X_{i} \in \{0,1,\ldots, S\}. $$

Similarly,

$$ d N_{t}= \textstyle\begin{cases} Y_{i} & \text{if day changes at time $t$,} \\ 0 & \text{on the same day}, \end{cases}\displaystyle \quad \text{and}\quad d K_{t}= \textstyle\begin{cases} Z_{i} & \text{if day changes at time $t$,} \\ 0 & \text{on the same day}, \end{cases} $$

where

7 $$ Y_{i}+Z_{i} \in \{0,1,2,\ldots,I\}. $$

The expected values of the above expressions can be expressed (see Lamberton and Lapeyre [27] and Karatzas and Shreve [31]) as

8 $$ \gamma t=\sum_{i=1}^{t}E[X_{i}],\qquad \lambda t=\sum_{i=1}^{t}E[Y_{i}] \quad \text{and}\quad \eta t=\sum_{i=1}^{t}E[Z_{i}], $$

with the following quadratic variations (see Karatzas and Shreve [31], p. 31)

9 $$ \langle M_{t}-\gamma t\rangle =\gamma t,\qquad \langle N_{t}-\lambda t\rangle=\lambda t \quad\text{and}\quad \langle K_{t}- \eta t\rangle =\eta t. $$

Moreover, the random processes $M_{t}-\gamma t$, $N_{t}-\lambda t$, and $K_{t}-\eta t$ are martingales (see Shreve [30]).

### Existence of the solution of the stochastic model

Here, we show the stability of the stochastic model and investigate an inequality managing of which leads to decrease of the number of COVID-19 patients.

#### Theorem 3

*For initial positive values* $S(0)$, $I(0)$, *and* $R(0)$ *in formulation* (3), *there exists a unique nonnegative solution* $(S(t), I(t), R(t))$, $t>0$, *with probability one*.

#### Proof

As all the coefficients in stochastic system (4) are locally Lipschitz continuous (see Mao [32]), there exists a unique maximum local solution $(S(t), I(t), R(t))$ on $t \in [0, T_{e})$, where $T_{e}$ is the explosion time.

To show that the solution is global, we will show that the explosion time $T_{e}=\infty $ a.s. Otherwise, we suppose that there is some bounded time such that the solution $(S(t), I(t), R(t))$ cannot explode to infinity. Let *N* be a large positive number such that the initial values $S(0)$, $I(0)$, and $R(0)$ belong to the interval $[\frac{1}{N}, N]$. For each integer $n\geq N$, let us define the sequence of stopping times

$$\begin{aligned} T_{n} = \inf_{t} \biggl\{ t \in [0, T_{e}): \min \bigl\{ S(t), I(t), R(t)\bigr\} \leq \frac{1}{n} \text{ or } \max \bigl\{ S(t), I(t), R(t)\bigr\} \geq n \biggr\} , \end{aligned}$$

and let $\inf (\emptyset )= \infty $. Note that the above sequence increases and denote $T_{\infty }= \lim_{n \rightarrow \infty }T_{n}$; then $T_{\infty }\leq T_{e}$ a.s.

If $T_{\infty }=\infty $ a.s., then also $T_{e}=\infty $ a.s. Thus all the values ($S(t)$, $I(t)$, and $R(t)$) are positive a.s. for all *t*. Otherwise, suppose there exists a pair $(\overline{t}, \varepsilon )$, $\overline{t}>0$ and $0<\varepsilon <1$, such that the probability $P\{T_{\infty }\leq \overline{t}\}>\varepsilon $. Thus there exists an integer $n_{1}\geq N$ such that the probability $P\{T_{\infty }\leq \overline{t}\}\geq \varepsilon $ for all integers $n_{1}\geq N$.

Next, let us define the smooth function $W:\mathbb{R}^{3}_{+}\rightarrow \mathbb{R_{+}}$ as

10 $$ W(S,I,R)= \bigl(S-1- \ln (S) \bigr)+\bigl(I-1-\ln (I)\bigr)+ \bigl(R-1-\ln (R)\bigr). $$

Using Itô’s formula (see Karatzas and Shreve [31]), we calculate

11 $$\begin{aligned} dW(S,I,R) ={}& W_{s}\,dS+W_{i} \,dI+W_{r}\,dR+\frac{1}{2} \bigl(W_{ss}\,d\langle S \rangle +W_{ii}\,d\langle I\rangle +W_{rr}\,d \langle R\rangle \bigr) \\ ={}& \biggl(1-\frac{1}{S} \biggr)\,dS+ \biggl(1-\frac{1}{I} \biggr) \,dI+ \biggl(1-\frac{1}{R} \biggr)\,dR \\ &{}+\frac{1}{2} \biggl(\frac{1}{S^{2}}\,d\langle S\rangle + \frac{1}{I^{2}}\,d\langle I\rangle+ \frac{1}{R^{2}}\,d\langle R\rangle \biggr), \end{aligned}$$

where $W_{\cdot }$ and $W_{\cdot \cdot }$ are the first- and second-order partial derivatives of *W*, with respect to the space variable respectively, and $\langle S\rangle $, $\langle I\rangle $, and $\langle R\rangle$ are the quadratic variations.

Using (3), (4), and (9), we calculate

12 $$\begin{aligned} dW(S,I,R) ={}& \biggl[a-\mu (S+R)-\frac{a}{S}+2 \mu + \frac{\gamma }{2S^{2}}+\frac{\gamma +\lambda +\eta }{2I^{2}}+ \frac{\eta }{2R^{2}} \biggr]\,dt \\ &{}-I \biggl(\frac{1}{S}-\frac{1}{I} \biggr)\,dM_{t}- \biggl(1-\frac{1}{I} \biggr)\,dN_{t}- \biggl(\frac{1}{I}+ \frac{1}{R} \biggr)\,dK_{t}. \end{aligned}$$

Using the martingale property of $M_{t}-\gamma t$, $N_{t}-\lambda t$,and $K_{t}-\eta t$, this stochastic equation becomes

$$\begin{aligned} dW = {}&LW(S,I,R)\,dt-I \biggl(\frac{1}{S}-\frac{1}{I} \biggr) \,d(M_{t}- \gamma t)\\ &{}- \biggl(1-\frac{1}{I} \biggr) \,d(N_{t}-\lambda t)- \biggl( \frac{1}{I}+\frac{1}{R} \biggr)\,d(K_{t}-\eta t), \end{aligned}$$

where

13 $$\begin{aligned} LW(S,I,R) ={}& a-\mu (S+R)-\frac{a}{S}+2\mu + \frac{\gamma }{2S^{2}}+ \frac{\gamma +\lambda +\eta }{2I^{2}}+\frac{\eta }{2R^{2}} \\ &{}+\gamma I \biggl(\frac{1}{S}-\frac{1}{I} \biggr)+\lambda \biggl(1- \frac{1}{I} \biggr)+ \eta \biggl(\frac{1}{I}+ \frac{1}{R} \biggr) \\ \leq {}& q+\frac{\gamma }{2S^{2}}+\frac{\gamma +\lambda +\eta }{2I^{2}}+ \frac{\eta }{2R^{2}}+ \frac{\gamma I}{S}+\eta \biggl(\frac{1}{I}+ \frac{1}{R} \biggr) \end{aligned}$$

with $q=a+2\mu + \lambda $.

Further, as the positive space variables *S*, *I*, and *R* represent the numbers of individuals, we have $\frac{I}{S}\leq 1$, $\frac{1}{I}\leq 1$, and $\frac{1}{R}\leq 1$. Using this, we write

14 $$ LW(S,I,R) \leq a+2\mu + 3\gamma +2\lambda +4\eta. $$

Thus by Theorem 2.2 in [33] we deduce that $T_{\infty }=\infty $, which completes the proof. □

Now we come to the inequality among some parameters of the stochastic model, managing of which leads to decrease of the number $I(t)$ of the infected COVID-19 patients.

#### Theorem 4

*The expected value of the infected class* $I(t)$ *decreases with time*, *that is*, $\frac{d}{dt}E[I(t)]\leq 0$ (*or* $I(t)$ *is supermartingale*) *if*

15 $$ \gamma I(0)\leq \lambda +\eta. $$

#### Proof

From (3) we write

$$\begin{aligned} (1-M_{t})I(t)=I(0)-N_{t}-K_{t}. \end{aligned}$$

Applying mathematical expectation to both sides and simplifying, we obtain

$$ E\bigl[I(t)\bigr]=\frac{I(0)-(\lambda +\eta )t}{1-\gamma t}, $$

and differentiating with respect to *t*, we find

$$\begin{aligned} \frac{d}{dt}E\bigl[I(t)\bigr]= \frac{\gamma I(0)-\lambda -\eta }{(1-\gamma t)^{2}}. \end{aligned}$$

Using inequality (15), we get the result. □

Note that in this work the parameter *γ* is calculated from $M_{t}$ by using equations (5) and (8), which is replaced by transmission and contact rate parameters. These parameters decrease by applying lock down, using a sanitizer, washing hands, and keeping social distance and awareness campaign. Moreover, *η* is calculated from $K_{t}$ replaced by the recovery term, and we know that recovery terms increase through strong immunity system of the body and good treatment. Decrease of *γ* and increase of *η* strick inequality (15). The strictness of (15) leads to control of disease COVID-19.

A controlling strategy is optimal if it decreases the value of $I(t)$ and strick inequality (15).

Finally, we use relations (5) in $I(t)$ and graph $I(t)$ using the COVID-19 data of USA from first April to 19th July, 2020 taken from [34]. We also calculate the values of *γ*, *λ*, and *η* to verify Theorem 4.

The following table shows the values of *γ*, *λ*, and *η* for April, May, June, and July, 2020, in case of COVID-19 data of USA [34].

In Table 1 the parameter *γ* is calculated from

$$ \gamma t=\sum_{i=1}^{t} X_{i} \times \frac{X_{i}}{\text{Total susceptibles at $(i-1)$th day}}. $$

Similarly, the parameters *λ* and *η* are calculated from

$$ \lambda t=\sum_{i=1}^{t} Y_{i} \times \frac{Y_{i}}{\text{Total active cases at $(i-1)$th day}} $$

and

$$ \eta t=\sum_{i=1}^{t} Z_{i} \times \frac{Z_{i}}{\text{Total active cases at $(i-1)$th day}}. $$

**Table 1:** Values of the stochastic parameters calculated from data of USA during COVID-19 for four months

Month	*γ*	*λ*	*η*
April	2.794370191	8.161600786	83.71889805
May	1.844039647	2.211604232	348.0864949
June	2.820176949	0.48994873	318.1660605
July	11.76093832	0.325367682	706.6299277

From the Table it is clear that the condition of Theorem 4 is not satisfied for every month, and therefore the value of $I(t)$ increases (see the data of the months of April, May, June, and July 2020 in [34]).

## Conclusion

**Figure 3:**
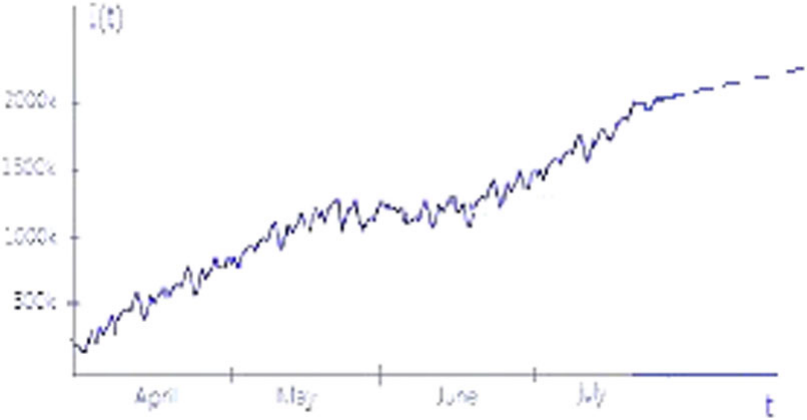
The sketch of Infected class of coronavirus

In this work, we analyze a stochastic model on the spread and control of coronavirus. First, we considered an SIR model to understand the spread and control of current infectious disease, then investigated the stability of the proposed model. Further, we investigated an inequality managing of which leads to decrease of the number of infected individuals. After this, we presented the nonnegative unique strong solution of the stochastic model and showed an inequality among some parameters of the model through martingale theory, managing of which leads to minimizing the number of COVID-19 patience. We found that decreasing the number of susceptible individuals, transmission, and contact parameters and increasing the recover parameter strick the inequalities. We found that applying lock down, use of sanitizer, washing hands, keeping social distance and awareness campaign decrease the value of susceptible individuals, transmission, and contact parameters, whereas a strong immunity system of the body and good treatment increase the value of recover parameter. We concluded that managing these terms leads to dropping the number of COVID-19 patients. The results are verified through the data of USA and shown in Fig. 3, and the managing parameters are calculated in the Table 1. Analysis of the results leads to understanding the flow and control of coronavirus in a clear way.
